# IMM47, a humanized monoclonal antibody that targets CD24, exhibits exceptional anti-tumor efficacy by blocking the CD24/Siglec-10 interaction and can be used as monotherapy or in combination with anti-PD1 antibodies for cancer immunotherapy

**DOI:** 10.1093/abt/tbad020

**Published:** 2023-09-09

**Authors:** Song Li, Dianze Chen, Huiqin Guo, Yanan Yang, Dandan Liu, Chunmei Yang, Xing Bai, Wei Zhang, Li Zhang, Gui Zhao, Xiaoping Tu, Liang Peng, Sijin Liu, Yongping Song, Zhongxing Jiang, Ruliang Zhang, Jifeng Yu, Wenzhi Tian

**Affiliations:** ImmuneOnco Biopharmaceuticals (Shanghai) Inc., Shanghai 201203, China; ImmuneOnco Biopharmaceuticals (Shanghai) Inc., Shanghai 201203, China; ImmuneOnco Biopharmaceuticals (Shanghai) Inc., Shanghai 201203, China; ImmuneOnco Biopharmaceuticals (Shanghai) Inc., Shanghai 201203, China; ImmuneOnco Biopharmaceuticals (Shanghai) Inc., Shanghai 201203, China; ImmuneOnco Biopharmaceuticals (Shanghai) Inc., Shanghai 201203, China; ImmuneOnco Biopharmaceuticals (Shanghai) Inc., Shanghai 201203, China; ImmuneOnco Biopharmaceuticals (Shanghai) Inc., Shanghai 201203, China; ImmuneOnco Biopharmaceuticals (Shanghai) Inc., Shanghai 201203, China; ImmuneOnco Biopharmaceuticals (Shanghai) Inc., Shanghai 201203, China; ImmuneOnco Biopharmaceuticals (Shanghai) Inc., Shanghai 201203, China; ImmuneOnco Biopharmaceuticals (Shanghai) Inc., Shanghai 201203, China; ImmuneOnco Biopharmaceuticals (Shanghai) Inc., Shanghai 201203, China; Department of Hematology, First Affiliated Hospital of Zhengzhou University, Zhengzhou 450051, China; Department of Hematology, First Affiliated Hospital of Zhengzhou University, Zhengzhou 450051, China; ImmuneOnco Biopharmaceuticals (Shanghai) Inc., Shanghai 201203, China; Department of Hematology, First Affiliated Hospital of Zhengzhou University, Zhengzhou 450051, China; Henan International Joint Laboratory of Nuclear Protein Gene Regulation, Henan University College of Medicine, Kaifeng 475004 Henan, China; ImmuneOnco Biopharmaceuticals (Shanghai) Inc., Shanghai 201203, China

**Keywords:** CD24, humanized monoclonal antibody IMM47, CD24/Siglec-10 interaction, cytokine secretion, cancer immunotherapy

## Abstract

This study evaluates the anti-tumor mechanism of IMM47, a humanized anti-CD24 mAb. Biolayer interferometry, ELISA and flow cytometry methods were used to measure the IMM47 binding, affinity, ADCC, ADCP, ADCT and CDC activities. *In vivo* therapeutical efficacy was measured in transplanted mouse models. IMM47 significantly binds granulocytes but not human erythrocytes and blocks CD24’s ability to bind to Siglec-10. IMM47 has strong ADCC, ADCT and ADCP activity against REH cells. IMM47’s *in vivo* pharmacodynamics showed that IMM47 has strong anti-tumor effects in human siglec-10 transgenic mouse models with a memory immune response. IMM47 also has powerful synergistic therapeutic efficacy when combined with Tislelizumab, Opdivo and Keytruda, by blocking CD24/Siglec-10 interaction through macrophage antigen presentation with strong ADCC, ADCP, ADCT and CDC activities and with a safe profile. IMM47 binding to CD24 is independent of N-glycosylation modification of the extracellular domain.

## Teaser

Statement of Significance: Novel humanized mAb MM47 has excellent anti-tumor efficacy by blocking CD24/Siglec-10 interaction with strong antibody-dependent cellular cytotoxicity, antibody-dependent cellular phagocytosis , antibody-dependent cellular trogocytosis and complement dependent cytotoxicity activities with a safe profile and has synergetic therapeutic efficacy when combining with programmed death-1 antibodies in cancer immunotherapy. Its binding to CD24 is independent of N-glycosylation modification of the extracellular domain.

## INTRODUCTION

CD24, a small, highly glycosylated protein that is overexpressed in many solid malignancies [1–7], has been considered a cancer biomarker [8, 9], while Siglec-10 is overexpressed in tumor-associated macrophages [10]. CD24 can interact with Siglec-10 on innate immune cells to reduce inflammatory responses to several disorders such as infection [11], sepsis [12], liver injury [13] and chronic graft versus host disease [14].

The binding of CD24 to Siglec-10 can activate the inhibitory SHP-1 and/or SHP-2 phosphatases, thereby inhibiting TLR-mediated inflammation and macrophage-induced cellular engulfment [15–17]. CD24 is a promising target for cancer immunotherapy, which has recently been recognized as one of the most effective approaches in the treatment of many tumors [10, 18–21]. Despite minimal efficacy data, monoclonal antibodies against CD24 have shown clinical safety and tolerability in two clinical trials [21, 22]. CD24Fc, a recombinant fusion protein that stimulates the CD24/Siglec-10 pathway, has been studied in patients with melanoma or advanced solid tumors [23]. However, two related clinical trials (NCT04552704, NCT04060407) have been terminated or withdrawn. We discuss our findings on the anti-tumor mechanism of IMM47, a humanized monoclonal antibody (mAb) targeting CD24, which inhibits CD24/Siglec-10 interaction via macrophage antigen presentation while increasing NK cell cytokine release.

## METHODS

### MATERIALS AND METHODS

#### Animals, cell lines and healthy human donors

Severe combined immunodeficiency (SCID) mice and C57BL/6 mice were purchased from Charles River and Shanghai Model Organisms, respectively. All animal procedures were performed in compliance with Institutional Animal Care and Use Committee (IACUC) guidelines and approved by the Institutional Animal Care and Use Committee of Shanghai Model Organisms Center, China. *In vivo* anti-tumor responses were evaluated using cancer cell line-derived transplantation. All animal experiments were repeated at least three times to ensure the results were reliable. Details of the procedures were described in the supplementary file. REH (cat# tchu131), Hela (cat# tchu187), MCF-7 (cat# tchu74), HCC1954 (cat# tchu245), 293T (cat# SCSP-502), Raji (cat# tchu44), Jurkat (cat# tchu123), THP-1 (cat# tchu57) and Jeko-1(cat# tchu194) were purchased from the cell bank of the Chinese Academy of Sciences. All cell lines were cultured under standard conditions and harvested at the logarithmic growth stage.

Peripheral blood samples were collected from healthy adult donors with written informed consent.

#### Construction and expression of mAb IMM47

IMM47 is a humanized mouse anti-human CD24 mAb that was developed with standard recombinant monoclonal antibody technology by using the in-house generated human CD24 antigen as the immunogen. The sequences of CD24 for humans (P25063), chimpanzees (A0A2J8PUR5), cynomolgus monkeys (I7GKK1), mice (P24807), rats (Q07490), pigs (K7GMK8) and dogs (E2R960) were collected from UniProtKB databases. IMM47 mAb proteins were purified with protein A affinity column chromatography. The purity of these fusion proteins was evaluated by size-exclusion high-performance liquid chromatography.

IMM47 was generated by the method described previously in the patent (CN113831412A) by ImmuneOnco Biopharmaceuticals (Shanghai) Inc. [24–26].

#### The determination of affinity between anti-CD24 antibody and antigen was measured using biolayer interferometry (BLI)

Kinetic analyses of IMM47 were performed using a biomolecular analyser (Access Medical Systems, Gatorbio). Analysis was carried out by selecting the kinetics assay mode, using Q buffer as the experimental buffer, and using a human Fc probe as the experimental probe. Each cycle includes baseline, loading (1 μg/ml, CD24-his), baseline, association (IMM47, starting 100 nM with six concentration gradients at two times dilution), dissociation, and regeneration. Setting the temperature of the instrument to 25 °C.

#### Analysis of binding affinity with CD24 target antigens from different species and with different CD24 mutants by enzyme-linked immunosorbent assay (ELISA)

The ELISA method was used to measure the binding affinity of IMM47 to CD24 target antigens from different species and to different CD24 mutants. Briefly, different species CD24 and different in-house developed CD24 mutant proteins, including CD24 (WT), mFc Tag, CD24 (N10A), mFc Tag, CD24 (N26A), mFc Tag and CD24 (N10A/N26A) and mFc Tag, were coated overnight at 4 °C in each well of a 96-well microplate. After washing twice with PBS, the wells were blocked with 100 μl of PBS plus 3% (w/v) skim milk and incubated for 2 h at room temperature. The testing samples were diluted with 3% skim milk to 10 μg/ml. followed by three times gradient dilutions; thereafter, 100 μl/well samples were loaded and incubated at 37 °C for 1 h; after washing with 300 μl/well washing buffer, peroxidase-conjugated AffiniPure F(ab’)2 fragment goat anti-human IgG, Fc-γ fragment specific antibody (Jackson Immuno, Cat#109-036-098) diluted with 3% skim milk were added with 100 μl/well and incubated at 37 °C for 1 h; samples were washed with 300 μl/well washing buffer. A total of 100 μl/well of TMB solution (KPL, Cat# 51200050) were added and incubated for 15 min at room temperature in the dark. Then the reaction was stopped by adding 50 μl/well of termination solution, and the OD450 values were measured. The results were analysed by using GraphPad Prism software to draw a graph. The combination curves were made with four parameters.

#### CD24 target binding assay and binding ability with different glycosylation modifications of CD24 by flow cytometry

MCF-7, HCC1954 and Hela tumor cells were digested with trypsin and collected after centrifugation at 1000 rpm for 5 min. For different CD24 glycosylation modification assays, Reh cell suspensions with 2 μl of PNGase F (New England Bio Labs, Cat# P0710S), or Sialidase A (Prozyme, Cat# GK80040) were incubated at 37 °C for 24 h before the assay. Cells were collected at 1000 rpm for 5 min. Cells were washed with 0.5% BSA-PBS once, and cell density was set at 5 × 10^5^ cells/ml. Antibodies were diluted to the corresponding concentration with three-fold gradient dilutions. About 50 μl of cells and 50 μl of antibody dilution were added into each well of the 96-well U-shaped plates and incubated at 4 °C for 45 min. After incubation, wells were rinsed with 150 μl of the 0.5% BSA-PBS solution. About 100 μl of the 1:500 diluted anti-human IgG (Fc)–FITC (Sigma, Cat# F9512) was added and incubated at 4 °C for 45 min. After incubation, wells were rinsed with 150 μl of the 0.5% BSA-PBS; and120 μl of the 0.5% BSA-PBS was added. The cells were measured with flow cytometry. GraphPad Prism software was used to analyse the data, and the combination curves were drawn with four parameters. The same method was used to verify the binding capacity of CD24-negative 293T cells.

#### Competition assay of IMM47 with ML5 and SN3 antibodies binding to CD24 positive cells by flow cytometry

Reh tumor cells were collected at 1000 rpm for 5 min. Cells were washed with 0.5% BSA-PBS once, and cell density was set to 5 × 10^5^ cells/ml. Antibodies were diluted to the fixed concentrations (ML5 0.5 μg/ml and SN3 1.0 μg/ml) with 0.5% BSA-PBS. IMM47 antibody was diluted to 30 μg/ml followed by a 3-fold gradient dilution. 50 μl of cells and 50 μl of antibody dilution were added into each well of the 96-well U-shaped plates and incubated at 4 °C for 45 min. After incubation, wells were rinsed with 150 μl of the 0.5% BSA-PBS. A total of 100 μl of the diluted IMM47 were added and incubated at 4 °C for 45 min. After incubation, wells were rinsed with 150 μl of the 0.5% BSA-PBS solution. A total of 100 μl of the diluted anti-human IgG (Fc)-FITC (Sigma, Cat# F9512) was added and incubated at 4 °C for 45 min. After incubation, wells were rinsed with 150 μl of the 0.5% BSA-PBS was added. The cells were measured with flow cytometry. GraphPad Prism software was used to analyse the data, and the combination curves were drawn with four parameters.

#### Analysis of binding activity on peripheral blood mononuclear cells (PBMC), granulocytes, B cells from different species (human, monkey) and red blood cells (RBCs)

A total of 1200 μl of pre-warmed RBC lysing buffer and 200 μl whole blood samples (at a 1:6 ratio) were added into 1.5 ml centrifuge tubes and incubated in the dark at room temperature for 4–8 min. The samples were centrifuged for 3 min at 3000 rpm, and the supernatant was discarded. Then the samples were rinsed with 1 ml of 1% BSA-PBS and resuspended in 150 μl of 1% BSA-PBS, and the cell density was adjusted to 1 × 10^6^ cells/ml. Antibodies were diluted to 60 μg/ml, followed by three-fold dilution for 10 concentration gradients. A total of 50 μl of cell suspension and 50 μl of antibody dilution were added to each well of the 96-well U-shaped plates and incubated at 4 °C for 45 min. After incubation, wells were rinsed once with 150 μl of the 0.5% BSA-PBS solution. A total of 100 μl of diluted FITC-conjugated anti-human IgG (Fc) were added into each well. For the B cell binding experiment, a PE/Cy5 anti-human CD20 antibody (Biolegend, Cat# 302308) was added at the same time. The plates were incubated at 4 °C for 45 min. After incubation, wells were rinsed with 150 μl of the 0.5% BSA-PBS and then resuspended with 120 μl of the 0.5% BSA-PBS. Samples were then analysed by flow cytometry. The results were analysed by using GraphPad Prism software to draw a graph. The combination curves were drawn with four parameters.

For the RBCs assay, a total of 50 μl whole blood was washed twice with 10 ml of PBS at 2000 rpm for 5 min. RBCs are harvested, counted, and adjusted for density to 1 × 10^6^ cells/ml by adding 1% BSA-PBS. Human and CD24-humanized mouse RBCs were incubated with IMM47 and hIgG1-Fc at different concentrations, followed by staining with the anti-human IgG (Fc)-FITC secondary antibody (Sigma, cat# F9512). Cells were analysed by flow cytometry to measure the binding activity of IMM47.

#### Agglutination test of CD24 humanized mouse erythrocytes and human erythrocytes

To evaluate the erythrocyte agglutination ability of the humanized antibody IMM47, the erythrocyte agglutination assay was done with the standard test procedure. Human erythrocytes and CD24-humanized mouse erythrocytes were diluted to 1% in PBS. Different concentrations of IMM47 and hIgG1-Fc were incubated in the round bottom of 96-well plates at room temperature for 1–2 h. Hemagglutination is indicated by the presence of non-precipitated RBCs, which is murky in comparison to the punctate red spots of non-hemagglutinating RBCs.

#### Detection of IMM47 blocking the interaction between CD24 and the Siglec-10 protein

About 293 T-CD24 cells were cultured in DMEM with 10% fetal bovine serum (Gemini, Cat# 900-108) and 30 μg/ml puromycin (Sangon Biotech, Cat# A610593-0025). Cells were collected and washed once with PBS buffer. A total of 2 × 10^5^ live cells in 25 μl were added to each well. About 25 μl of diluted IMM47 antibodies, starting at 5 μg/ml followed by three-fold dilution concentrations, were added into each well and incubated at 4 °C for 45 min. About 50 μl of the diluted PE-labeled human Siglec-10 (Acrobiosystems, Cat# SI0-HP2E5) solution were added to each well and incubated at 4 °C for 45 min. Wells were rinsed with 200 μl of the PBS buffer three times and resuspended with 120 μl of the PBS buffer. Samples were detected by flow cytometry. GraphPad Prism software was used to analyse the data, and the combination curves were drawn with four parameters.

#### Antibody-dependent cell-mediated cytotoxicity (ADCC) assay

About 50 μl of MCF-7, HCC195 and Hela target cells labeled with carboxyfluorescein succinimidyl ester (CFSE) (Sigma, Cat# 21888), 50 μl of CD16a (158 V) overexpression NK92MI cell line (FcR-TANK, developed in-house) at a 1:2 ratio, and 100 μl of the diluted IMM47 antibodies starting at 40 nM or 4 μg/ml, followed by three times dilution for 12 concentration gradients, were added into each well and incubated at 37 °C with 5% CO_2_ for 4 h. After incubation, place the cell culture plate at room temperature for 10 min and return it to room temperature. About 20 μl of the diluted PI solution (Sigma, Cat# P4170) were added to each well with a final concentration of 5 μg/ml and mixed well. Samples were then analysed by flow cytometry, and the numbers of PI-positive staining cells were calculated. Calculation of ADCC intensity: Lysis% = (sample% PI positive cell—no antibody% PI positive cell)/(100—no antibody% PI positive cell) × 100%.

#### Antibody-dependent cellular phagocytosis (ADCP) assay

Macrophage induction and differentiation from PBMC: PBMCs were isolated from whole blood using lymphocyte isolation solution histopaque®-1077 (Sigma, Cat# 10771), and then monocytes were isolated by magnetic bead negative selection (easysep) ™ Human Monocyte Isolation Kit (Stemcell, cat# 19359). The isolated monocytes were then cultured with ImmunoCult™-SF Macrophage Medium (Stemcell, Cat# 10961) containing M-CSF (Stemcell, Cat# 78057.1) for at least 10 days to differentiate into macrophages.

Macrophage plate coating: The macrophage culture supernatant was discarded by pipetting and washed twice with PBS solution. Then the adhesive cells were digested with 0.25% trypsin for 5 min at room temperature. The cells were then gently pipetted, and the digestion process was stopped with macrophage medium after the cells fell off. The cells were then collected by centrifugation at 1000 rpm for 5 min. The cells were re-suspended and adjusted to a density of 2 × 10^5^/ml. After mixing, 1 μg/ml LPS (Sigma, cat# l4516) and 100 ng/ml IFN-r (Stemcell, cat# 78020.1) were added into each well at 100 μl per well of the 96-well plates. The plates were then cultured in an incubator at 37 °C with 5% CO_2_ for 2 days.

Dilution of CD24 blockers: CD24 blockers (IMM47, IMM01, and hIgG1-Fc) were diluted with 1640 complete culture medium to a concentration of 200 nM, followed by a four-fold dilution to eight concentration gradients.

CFSE target cells, CD47 blockers, and macrophages (with an E/T ratio of 1:2) were mixed and incubated at 37 °C with 5% CO_2_ for 2 h. The supernatant and free CFSE target cells were removed, and fluorescence microscope photos were taken to record the fluorescence intensity of macrophages. The plates were rinsed with 200 μl of the PBS buffer, and then flow cytometry was performed to analyse the green fluorescence intensity of macrophages. GraphPad Prism software was used to analyse the data, and the combination curves were drawn with four parameters.

A similar method was used to detect the blocking effect of ADCP activity against tumor cells (THP-1 cells) instead of macrophages, as described earlier.

The phagocytosis ratio was calculated with the following formula: phagocytosis (%) = experimental group phagocytosis (%)– blank control group phagocytosis (%).

#### Antibody-dependent cellular trogocytosis (ADCT) assay

About 100 μl of THP-1 cells were diluted to a concentration of 4 × 10^5^/ml and cultured with 200 ng/ml PMA at 37 °C with 5% CO_2_ for 6 h. Then 50 μl of 1640 complete culture medium containing IFN-g (20 ng/ml) were added and cultured for 18 h. About 50 μl of Reh cells at a concentration of 5 × 10^5^/ml were added to each well. About 50 μl of diluted IMM47, starting at 10 ug/ml with a four-fold dilution for nine concentration gradients, were added into each well and incubated for 2.5 h. About 100 μl of 100 ng/ml IMM47 was added to Reh cells and incubated for 30 min at 4 °C. After washing once, 100 μl of the FITC-conjugated anti-human IgG Fc were added and incubated at 4 °C for 30 min. Flow cytometry was used to detect the FITC fluorescence signal of Reh cells.

#### Complement-dependent cytotoxicity (CDC) assay

REH cells were incubated with different concentrations (starting at 400 nM followed by a four-fold dilution to eight concentration gradients) of IMM47, hIgG1-Fc and normal human serum complement (Quidel, Cat# A113) at 37 °C with 5% CO_2_ for 4 h, followed by staining with PI solution. The flow cytometry method was used to collect the cells, and the PI-positive staining cells were calculated. The calculation of CDC intensity was done by using the following formula: Lysis % = Experimental Sample Lysis %- No. Antibody Lysis %.

#### *In vivo* pharmacodynamic and mechanism of action study in mouse models

The standard procedure was used for all analogous model establishments, including MC38-hCD24 cells injected with C57 BL/6 wild-type mice, MC38-hCD24 cells inoculated with Siglec-10 transgenic C57 BL/6 mice, and MC38-hCD24/hPD-L1 cells inoculated with PD-1 transgenic C57 BL/6 mice. To summarize, tumor cells were collected at the logarithmic development stage, and the cell suspension was subcutaneously implanted on the right flank of the mouse at a dose of 100 μl per mouse, containing approximately 3.0 × 10^6^ tumor cells. When the average tumor volume reached ~ 100 mm^3^, the animals were divided into groups using a randomized grouping procedure according to the experimental protocol, and the pharmaceutical treatment began. Tumor volumes and body weight were measured three times each week, and clinical symptoms were evaluated and documented once daily. The tumor volume inhibition rate (TGI) and tumor size were calculated using the following formula:


$$ TGI=\left[1-\left(T{V}_t-T{V}_{initial}\right)/\left(C{V}_t-C{V}_{initial}\right)\right]\times 100\% $$

Where TV_t_ represents the tumor volume at each measurement in the treatment group; TV_inital_ represents the tumor volume of the treatment group at the time of grouping administration; CV_t_ represents the tumor volume at each measurement in the control group; and CV_initial_ represents the tumor volume of the control group at the time of grouping administration.

All heterologous models were performed with a similar method as described above, including MCF-7 cells inoculated with SCID-CB17 mice and Jeko-1 cells inoculated with SCID-CB17 mice.

#### Statistical analysis

SPSS and Graphpad Prism 8.0 software (San Diego, CA, USA) are used for statistical analysis, and the results are expressed as mean ± standard deviation. Statistically significant differences between different groups were analysed using the Student t-test. Kaplan–Meier survival curves were used to compare survival rates between different groups. *P* < 0.05 indicates a statistically significant difference.

## RESULTS

### M‌M47 binds to CD24 with high specificity and affinity

**Figure 1:**
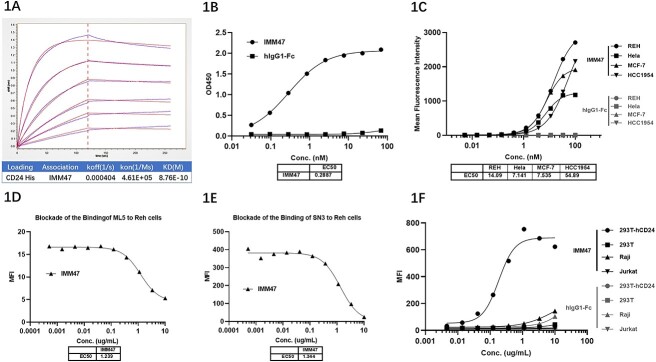
CD24 target binding activity. (A) Affinity (BLI) assay showed that the affinity between IMM47 and CD24 was 0.876 nM. (B) ELISA results showed that the EC50 of IMM47 binding to CD24 was 0.289 nM. However, hIgG-Fc did not bind to CD24 protein. (C) CD24 target binding to CD24 positive tumor cells by flow cytometry. EC50 of IMM47 binding to REH, Hela, MCF-7 and HCC1954 cells was 14.090, 7.141, 7.535 and 54.890 nM, respectively. However, hIgG-Fc did not bind to all cells. (D) At the fixed concentration of PE anti-hCD24 ML5 at 0.5 μL/sample plus serial dilution of IMM47, the EC50 was 1.239 μg/ml. The result demonstrated that IMM47 and ML-5 bind to the same target due to competition between them. 1E. At the fixed concentration of anti-hCD24 SN3 at 1 μg/ml plus serial dilution of IMM47, the EC50 was 1.344 μg/ml. The result demonstrated that IMM47 and SN3 bind to the same target due to competition between them. (F) The CD24 target binding specificity verification assay showed that IMM47 does not bind to CD24-negative cells (293T, Raji, Jurkat).

IMM47 mAb was constructed and produced using an in-house-developed CHO-K1 cell expression system. Affinity testing with the BLI assay showed that the affinity between IMM47 and CD24 was 0.876 nM (Fig. 1A). ELISA results showed that the half-maximal effective concentration (EC50) of IMM47 binding to CD24 was 0.289 nM (Fig. 1B). CD24 target binding assay by flow cytometry showed that the EC50 of IMM47 binding to REH, Hela, MCF-7 and HCC1954 cells were 14.09, 7.14, 7.54, and 54.89 nM, respectively (Fig. 1C). Blocking assays demonstrated that IMM47 competes with anti-CD24 mAbs (SN3, ML5) for binding, suggesting that IMM47 specifically binds to CD24 molecules (Fig. 1D and E). However, IMM47 does not bind to CD24-negative cells (293T, Raji, Jurkat) (Fig. 1F).

### The binding of IMM47 to CD24 is independent of glycosylation modifications

**Figure 2:**
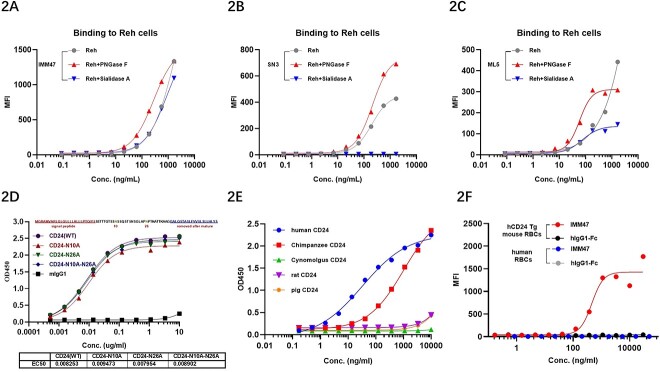
IMM47 binding to CD24 is independent of N-glycosylation and cross-reaction between different species’ CD24. (A-C) The extracellular domain of CD24 has 14 O-glycosylation and 2 N-glycosylation sites. N-glycosidase and sialidase-treated Reh cells had little effect on the affinity of IMM47 (A) and ML5 (C), and N-glycosidase-treated Reh cells even enhanced their binding ability; SN3 (B) can bind to Reh cells significantly, and the cells treated with sialidase cannot bind to SN3, indicating that sialylation of CD24 on Reh cells is crucial for SN3 binding. The result demonstrated that IMM47 binding to CD24 is independent of glycosylation modifications. (D) The extracellular domain’s N-glycosylation alterations had no effect on IMM47’s ability to bind to CD24. The result demonstrated that IMM47 binding to CD24 is independent of N-glycosylation modifications. (E) Analysis of cross binding activity between IMM47 and different species’ CD24 by ELISA. The result showed that IMM47 bind to CD24 protein of human and chimpanzee, and not for cyno monkey, rat and pig. (F) Binding activity of IMM47 to human RBCs or RBCs of mouse expressing human CD24, measured by FACS. The result showed that IMM47 did not bind to human RBCs but bind to RBCs of hCD24 Tg mouse.

**Figure 3:**
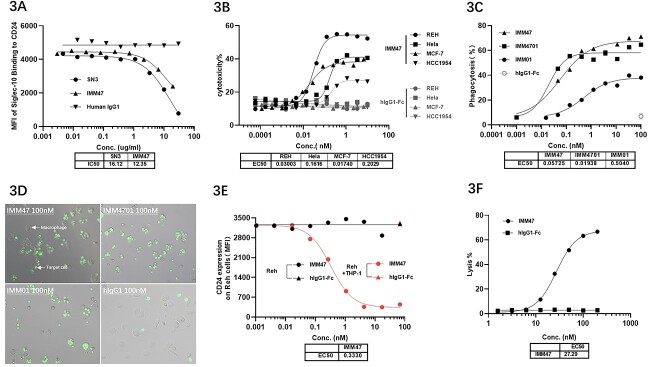
Mechanism of action study showed that IMM47 antibody had strong blocking, ADCC, ADCP, ADCT and CDC activities. (A) Mechanism of action study: CD24 target blocking activity results showed that the IMM47 antibody had a strong ability to block the binding of Siglec-10 and CD24. (B) Mechanism of Action Study: ADCC activity results showed that the EC50 of IMM47 for ADCC of REH, Hela, MCF-7 and HCC1954 is 0.030, 0.162, 0.017 and 0.202 nM, respectively. (C) Mechanism of action study: ADCP activities (PBMC monocyte-induced macrophages) The results showed that IMM47 can significantly induce the phagocytosis of macrophages against Reh cells. The EC50 was 0.057 nM, and the phagocytic activity was significantly stronger than IMM01 (SIRPα-Fc fusion protein). (D) ADCP activity of PBMC-derived macrophage was observed by fluorescent microscope. The results showed that IMM47 can strongly induce the phagocytosis than MM01 against Reh cells. (E) Mechanism of action study: The ADCT assay results showed that IMM47 has strong ADCT activity with an EC50 of 0.333 nM against REH. The downregulation of CD24 on the surface of tumor cells reduces Siglec-10/CD24 signal transduction and enhances the attack of immune cells on tumor cells. (F) Mechanism of action study: CDC activity results showed that the EC50 of IMM47 against REH cells is 27.29 nM.

CD24, a small and highly glycosylated protein, is attached to the cell membrane surface through a glycosyl-phosphatidylinositol anchor. The extracellular domain of CD24 only has 33 amino acids, but it has 14 O-glycosylation and 2 N-glycosylation sites. IMM47 binding to CD24 is independent of the N-glycosylation modifications in the extracellular domain. The affinity of IMM47 and ML5 was unaffected by the treatments with N-glycosidase and sialidase on Reh cells. The treatment of Reh cells with N-glycosidase even enhanced the binding ability to IMM47, SN3 and ML5 mAbs. SN3 mAb significantly binds to Reh cells but loses binding activity after treatment with sialidase, indicating that the sialylation of CD24 on Reh cells is crucial for the binding of SN3 (Fig. 2A–C). Furthermore, the extracellular domain’s N-glycosylation alteration had no effect on IMM47’s ability to bind to CD24 (Fig. 3D).

### IMM47 binds to the CD24 protein of humans and chimpanzees, not that of Cynomolgus monkeys, rats and pigs

Cross-binding homologous examination of CD24 target species revealed that human CD24 has high similarity with chimpanzees (96.97%) and low homology with Cynomolgus monkeys (45.45%). Cross-binding findings for CD24 target species revealed that IMM47 can only bind to human and chimpanzee CD24 but not to other species such as mice, rats, pigs, dogs and cynomolgus by ELISA and FACS assays (Fig. 2E and Fig. S1). Flow cytometry results for the IMM47 binding assay revealed that IMM47 does not bind to human erythrocytes (Fig. 2F), but robustly binds to human granulocytes and weakly binds to human B cells (Fig. S2). IMM47 can greatly increase erythrocyte agglutination in hCD24 transgenic mice but has no effect on human erythrocytes (Fig. S3).

### IMM47 blocks the interaction between CD24/Seglec-10 and kills tumor cells through mechanisms such as ADCC, ADCP, ADCT and CDC

Competitive binding assay demonstrated IMM47 blocking the interaction of CD24/Siglec-10 at a similar level of the anti-CD24 antibody SN3 (Fig. 3A). The stimulation of phagocytosis, complement activation and antibody-dependent cellular cytotoxicity (ADCC) can all be used for immune-mediated tumour cell killing. The EC50 of IMM47 ADCC against REH, Hela, MCF-7 and HCC1954 was found to be 0.030, 0.162, 0.017 and 0.202 nM, respectively (Fig. 3B). The measurement of ADCP activity in THP-1-induced macrophages revealed that the EC50s of IMM47 against REH and MCF-7 were 0.067 and 0.099 nM (Fig. S4), respectively. The ADCP activity of macrophages elicited by PBMC monocytes revealed that IMM47, with an EC50 of 0.057 nM, could better promote macrophage phagocytosis against Reh cells compared to IMM01 (a SIRPα-Fc fusion protein) (Fig. 3C and D). The trogocytosis experiment revealed that IMM47 can generate substantial trogocytosis of THP-1-induced macrophages against Reh cells, with an EC50 of 0.333 nM (Fig. 3E). Furthermore, the CDC assay results showed that the EC50 of IMM47 against REH cells was 27.29 nM (Fig. 3F).

### IMM47 demonstrates potent and robust anti-tumor efficacy as monotherapy or in combination with anti-PD1 antibodies for cancer immunotherapy

**Figure 4:**
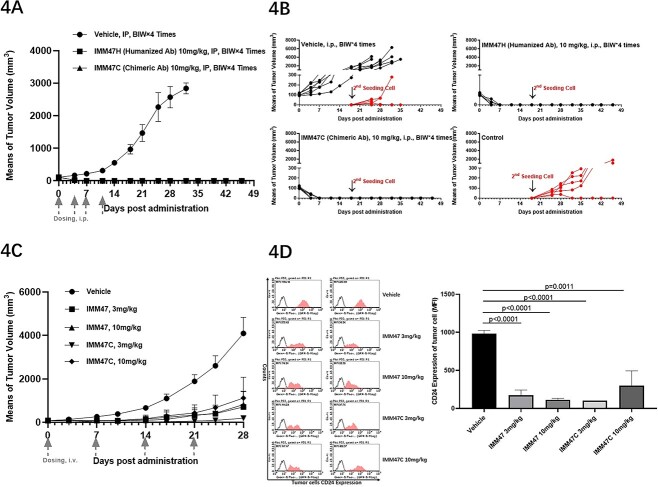
*In vivo* efficacy of IMM47 anti-tumor activities in transplanted mouse models. (A) *In vivo* efficacy assay: hSiglec-10 Tg C57BL/6 mice congenitally transplanted with MC38-hCD24. IMM47 showed strong anti-tumor activity on the homologous model of MC38-hCD24 cells inoculated into hSiglec-10 transgenic C57BL/6 mice, and the tumor in the treatment group was eliminated. (B) The mice in the treatment group were inoculated with tumor cells again, but the tumor cells could not form tumors, suggesting that a memory immune response was formed after treatment with IMM47. (C) *In vivo* efficacy in C57BL/6 mice congenitally transplanted with MC38-hCD24: IMM47 and IMM47C (chimeric Ab) showed strong anti-tumor activity in C57BL/6 wild-type mice inoculated with the MC38-hCD24 cell homologous model. There was no significant difference between the two groups. (D) The tumor cells CD24 expression were measured after IMM47 treatment by FACS. The tumor was stripped at the end of the experiment, and the cell suspension was prepared to detect the expression of CD24 on the surface of tumor cells. The CD24 expression on tumor cells in the treatment group mice was significantly downregulated and tumor growth was inhibited. IMM47 not only exhibited potent Fc-dependent effector functions to attack on tumor cells but also inhibited the Siglec-10/CD24 signal by downregulating CD24 expression of tumor cell.

Using hSiglec-10 Tg C57BL/6 mice congenitally transplanted with MC38-hCD24, our *in vivo* efficacy study showed that IMM47 had strong anti-tumor activity with a dose of 10 mg/kg, eliminating all tumors in the treatment group mice (Fig. 4A). After 20 days of treatment, three mice per group were euthanized, and their spleens were separated for analysis of M1 and M2 macrophages. The results show that spleen M1 and M2 macrophages in the IMM47 treatment group were significantly higher than those in the control group (Fig. S5). After stopping administration for 1 week, the remaining mice in the treatment group were inoculated with tumor cells again, but the tumor cells could not form tumors, suggesting that a memory immune response was established after IMM47 treatment (Fig. 4B). In hSiglec-10 transgenic C57BL/6 or wild-type mice inoculated with MC38-hCD24 cell syngeneic models, both demonstrated potent tumor growth inhibition, at a dose of 3 mg/kg, the CR were 5/6 and 4/6, respectively. (Fig. S6). In C57BL/6 wild-type mice inoculated with the MC38-hCD24 cell syngeneic model, the efficacy of the humanized antibody IMM47 and the chimeric antibody IMM47C was compared. IMM47 and IMM47C showed strong anti-tumor activity, and there was no significant difference between the two groups (Fig. 4C). The tumor was stripped at the end of the experiment, and the cell suspension was prepared to detect the expression of CD24 on the surface of tumor cells. The CD24 expression on tumor cells in the treatment group mice was significantly downregulated, and tumor growth was inhibited (Fig. 4D). IMM47 (IgG1 isotype) demonstrated a good tumor-killing effect at a dose of 3.0 mg/kg, while IMM4703 (IgG4 isotype) showed less anti-tumor activity even at a dose as high as 10 mg/kg, indicating the importance of Fc function resulting from the IgG1 isotype of IMM47 (Fig. S7). In the SCID mouse with the Jeko-1 tumor cell xenotransplantation model, IMM47 also exhibited strong dose-dependent anti-tumor activity (Fig. S8). Furthermore, in the SCID mouse with MCF-7 tumor cell xenotransplantation model, the combination of IMM47 and IMM01 had significantly better efficacy than any monotherapy (Fig. S9).

**Figure 5:**
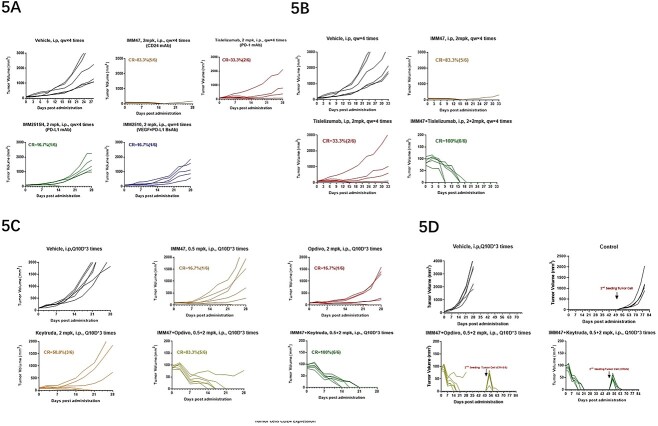
*In vivo* efficacy-IMM47 combined with PD-1 antibody (A). In the MC38-hCD24/hPD-L1 tumor cell congenitally transplanted PD-1 transgenic C57BL6 mouse model, IMM47 demonstrated the best anti-tumor activity with a complete response rate of 83%, while the Tislelizumab, IMM2515H (PD-L1 antibody) and IMM2510 (PD-L1 × VEGF BsAb) were 33.33%, 16.7% and 16.7%, respectively, at the same dose. (B) In the MC38-hCD24/hPD-L1 tumor cell congenitally transplanted PD-1 transgenic C57BL/6 mouse model, the combo of IMM47 with Tislelizumab had the best therapeutic effect, and all tumors in mice were eliminated. (C) In the MC38-hCD24/hPD-L1 tumor cell congenitally transplanted PD-1 transgenic C57BL/6 mouse model, the treatment efficacy of the combination of IMM47 and PD-1 antibodies Opdivo and Keytruda was better than that of each individual monoclonal antibody, and the tumor was removed in five of six mice in combination with Opdivo and all six mice in combination with Keytruda. (D) 5 weeks after the discontinuation of combined treatment with IMM47 and Opdivo and Keytruda, reinoculation of the same cancer cells into the mice, tumor growth was quickly and completely eliminated, suggesting tumor-specific immune response has been established.

In the comparison of *in vivo* efficacy among IMM47, IMM2515H (anti-PD-L1 antibody), IMM2510 (VEGF × PD-L1 bispecific Ab) and Tislelizumab all at a dose of 2 mg/kg, IMM47 demonstrates the best anti-tumor activity with a complete response rate of 83%, much better than the best of the rest, Tislelizumab, with a CR rate of 33% (Fig. 5A). The *in vivo* pharmacodynamic results of the combination of IMM47 and PD-1 antibodies showed that in the MC38-hCD24/hPD-L1 tumor cells of the homologous transplanted PD-1 transgenic C57BL/6 mouse model, the combination of IMM47 and the PD-1 antibody Tislelizumab had the best therapeutic effect, and all tumors in mice were eliminated (Fig. 5B). Further evaluation of the combination of IMM47 and the PD-1 antibodies Opdivo and Keytruda showed significant but similar anti-tumor activity at a relatively lower dose, with Keytruda showing better therapeutic efficacy (CR = 50%) than IMM47 (CR = 16.7%) and Opdivo (CR = 16.7%). While the combination of IMM47 with either Opdivo or Keytruda at a comparable dose demonstrates potent and robust anti-tumor activity, with a CR rate of 83.3% and 100%, respectively (Fig. 5C). Most intriguingly, upon reinoculation of the same cancer cells into the mice pretreated with a combination of IMM47 and anti-PD-1 antibodies after 5 weeks of discontinuation, tumor growth was quickly and completely eliminated, suggesting a tumor-specific immune response has been established. While the tumor cells in the control group grew normally (Fig. 5D).

## DISCUSSION

Through the overexpression of anti-phagocytic surface proteins, also known as the “don’t eat me” signal, such as CD47 [27], programmed cell death ligand 1 (PD-L1) [28], and the beta-2 microglobulin subunit of the major histocompatibility class I complex (B2M) [29], cancer cells can evade clearance by macrophages. Several malignancies have shown success with therapeutic monoclonal antibodies that block the interaction of “do not eat me” signals with their macrophage-expressed receptors [27–29].

As a strong anti-phagocytic “don’t eat me” signal, CD24 can help cancer cells avoid being attacked by macrophages that express Siglec-10 [4]. CD24 is crucial to the development of tumors. Several oncogenic signaling pathways, including Src/STAT3, EGFR, HER2, MAPK, AKT/mTOR, WNT/-catenin and miRNA-related pathways, have been associated with changes in tumor cell surface CD24 expression [8]. The growth of tumor cells and the increase of CD24 in the cytoplasm may be related [17]. Different solid tumors and hematologic cancers have been shown to overexpress CD24 with an anti-phagocytic signal [4, 8, 18]. In preclinical investigations, monoclonal antibody inhibition of the CD24-Siglec-10 interaction for cancer immunotherapy has gained increasing interest and demonstrated encouraging outcomes [4].

In order to assess the role of CD24-Siglec-10 signaling in regulating the immune response mediated by macrophages and the therapeutic potential of anti-CD24 monoclonal antibodies in cancer immunotherapy, we generated the humanized mAb IMM47 that targets CD24 using an internal CHO-K1 cell expression method.

IMM47 selectively binds CD24-positive cells but not CD24-negative cells. More crucially, our results showed that the human CD24 shares little in common with other species, such as the cynomolgus monkey, mouse, rat, pig, dog and chimpanzee, and more homology with chimps. Siglec-10 interacts with the heavily sialylated form of CD24, and surface desialylation significantly inhibits Siglec-10-Fc binding to MCF-7 cells, according to studies [2–4]. Our research showed that IMM47 can bind to CD24 without the extracellular domain being modified by N-glycosylation. This suggests that Siglec-10 can recognize both protein and sialic acid ligands, indicating that it likely has access to additional ligands in addition to CD24.

In many solid tumor models utilized in preclinical investigations, mAbs targeting CD24 have been employed either with or without chemotherapy. The ALB9 mAb blocked human urothelial carcinoma cells that overexpressed CD24 from spreading to the lungs, but lung colonization soon returned after the treatment was terminated [17]. SWA11, another anti-CD24 mAb, enhanced the anti-tumor effects of doxorubicin, oxaliplatin, 5-fluorouracil, irinotecan and paclitaxel, among other chemotherapy agents [18]. Correlations between the antiproliferative effect and CD24 expression levels were observed and dual CD24 inhibition with SWA11 and ML-5 prevented the spread of CD24^+^ pancreatic cancer cell lines [19]. Pre-treatment with SWA11 increased the anticancer efficacy of gemcitabine *in vivo*, mostly by promoting macrophage infiltration and reducing angiogenesis [20].

Two clinical trials established the clinical safety and acceptability of employing mAbs to inhibit CD24 as a novel and promising strategy for treating breast and ovarian cancer [4, 8]. Siglec-E’s sialylation-dependent recognition of CD24 results in the recruitment of SHP-1 and the suppression of metaflammation, therefore preventing metabolic syndrome. The CD24/Siglec-E axis, which serves as an innate immunological checkpoint against metaflammation and metabolic disorders, is a potential therapeutic target for metabolic diseases [17]. A first-in-human study of CD24-Fc (NCT02650895) provides evidence for the significance of this pathway in human lipid metabolism and inflammation.

Additionally, the G7 mAb has shown anti-tumor efficacy. G7 mAb displayed an anti-tumor effect when coupled with cetuximab, perhaps by disrupting STAT3 signaling [17, 18]. Preclinical studies [19] also investigated the potential of combining CD24 and CD47 for the treatment of glioblastoma. Furthermore, early studies on the development of a bispecific antibody against the NK receptor ligands MICA and G7 have produced promising results [20, 21]. The effectiveness of CD24-targeting recombinant antibodies has also been investigated. Single-chain variable fragments (scFvs) selected from a comprehensive RNA library of lymphocytes from breast cancer patients showed excellent selectivity for CD24 and CD44 when combined with epirubicin [22]. As an alternative to targeting CD24 in malignancies, ScFvs have FDA approval for clinical use, including blinatumomab for acute lymphoblastic leukemia [17].

The discovery that CD24 overexpression was associated with cancer and had demonstrated therapeutic promise from preclinical investigations [4, 8, 17–20] led to the development of IMM47. We postulated that CD24 blockage with a monoclonal antibody targeting CD24 could have anti-tumor therapeutic benefits while preserving a good safety profile and synergistic therapeutic effects when used in conjunction with other anti-tumor antibodies. According to our research, IMM47 only attaches to CD24-positive cells and dissociates from CD24-negative cells. Furthermore, the extracellular domain’s N-glycosylation alteration had no effect on IMM47’s ability to bind to CD24. N-glycosidase or sialidase treatment of Reh cells improved their capacity to bind to IMM47 mAb. The results of the cross-binding research using flow cytometry demonstrated that IMM47 can only bind to CD24 from humans and chimpanzees and cannot bind to CD24 from other species, confirming the high homology of human CD24 with chimpanzees and the poor homology with other species. This demonstrates the value of species specificity in anti-human CD24 monoclonal antibodies and paves the way for additional animal model-based research in the future.

CD24, a powerful anti-phagocytic “don’t eat me” signal, can shield cancer cells from attack by macrophages that are Siglec-10-expressing. The CD24-Siglec-10 signal is blocked by an anti-CD24 mAb, which is regarded as a new kind of innate immune checkpoint inhibitor. It demonstrated promise for the treatment of breast cancer and ovarian cancer by modulating anti-tumor immunity [4]. IMM47 exhibits excellent anti-tumor efficacy, according to the results of our *in vivo* drug efficacy assay using hSiglec-10 Tg C57BL/6 mice congenitally implanted with MC38-hCD24. Five out of six mice’s tumors were completely removed at a dose of 3 mg/kg. Additionally, IMM47 and anti-PD-1 antibodies exhibit substantial therapeutic synergy, demonstrating that IMM47 has a wide range of potential in cancer immunotherapy, whether used alone or in conjunction with PD-1 antibodies.

**Figure 6:**
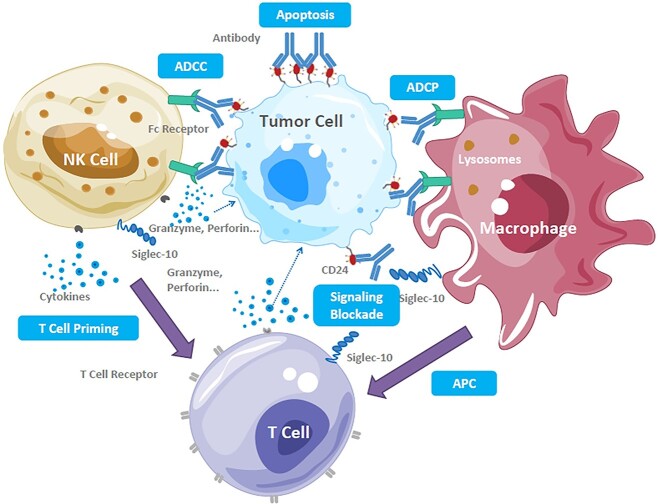
Structure of IMM47 and its mechanism of action for anti-tumor activities IMM47 binds to CD24 on the surface of tumor cells, promoting the activation of macrophages and NK cells through ADCP and ADCC and further activating the T cell immune response by the direct blocking of suppressive CD24/Siglec-10 interaction, as well as through macrophage antigen presentation and priming cytokines released by NK cells, inducing an all-around immune response.

Our findings indicated that IMM47’s mode of action involves binding to CD24 on the surface of tumor cells, which promotes macrophage activation. A bispecific antibody against the NK receptor ligands MICA and G7, Rg7S-MICA, was reported to activate NK cells and CD24+ human HCC cells, resulting in NK cell-mediated cytolysis *in vitro*. Additionally, Rg7S-MICA successfully attracted NK cells and stimulated the release of cytokines in CD24^+^ HCC-bearing nude mice to produce superior anti-tumor activity [37, 38]. By combining our data with previously published study findings, we deduce that IMM47 can directly disrupt the suppressive CD24/Siglec-10 interaction to directly stimulate the T cell immune response while also directly activating NK cells through ADCP and ADCC. This suggests that IMM47 may be highly valuable in its prospective clinical applications. Through macrophage antigen presentation and cytokines generated by NK cells, IMM47 can exert anti-tumor activity and trigger a thorough immune response (Fig. 6). IMM47 can therefore offer a fresh method for the therapeutic use of tumor-targeted immunotherapy. The induction of ADCP/ADCT and ADCC by macrophages and NK cells, respectively, can be one of the mechanisms of potential therapeutic effects by targeting CD24/Siglec-10 in addition to the direct blockade of the CD24/Siglec-10 signaling pathway to reduce the tumor cell “don’t eat me” signal and induce apoptosis. Additionally, CD24 may facilitate T cell activation and cytokine production via macrophage antigen presentation.

Therefore, our findings support the hypothesis that anti-CD24 immunotherapy can inhibit the CD24/Siglec-10 signaling pathway and destroy the tumor cells through a variety of mechanisms, such as by blocking tumor cell “don’t eat me” signals, inducing apoptosis in tumor cells, triggering ADCP from macrophages and ADCC from NK cells. However, more mechanism studies need to be investigated further.

## CONCLUSION

Our findings demonstrate that the humanized anti-CD24 mAb IMM47 exhibits exceptional anti-tumor activity by blocking the CD24/Siglec-10 interaction through macrophage antigen presentation. The extracellular domain’s N-glycosylation alteration has no effect on IMM47’s ability to bind to CD24. The *in vitro* assays revealed that IMM47 exhibits significant ADCC, ADCP, ADCT and CDC activities. IMM47 exhibits potent anti-tumor efficacy in transgenic mouse models, according to the findings of an *in vivo* pharmacodynamics analysis, and a memory immune response is established following therapy. Additionally, an *in vivo* pharmacodynamics assay of IMM47 in combination with different PD-1 antibodies revealed that IMM47 exhibits synergistic therapeutic efficacy when combined with Tislelizumab, Opdivo and Keytruda. This suggests that IMM47 has significant potential for use in the clinical setting for cancer immunotherapy, either alone or in combination with other immune checkpoint inhibitors. Finally, IMM47 avoids the adverse effects displayed by some anti-CD47 antibodies because it does not bind to human RBCs and has a high safety profile. The clinical trial application for IMM47 has been submitted in Australia, the United States and China, and the efficacy data of human clinical trials are yet to be determined.

## Supplementary Material

Fig_tbad020_S1_tbad020

Fig_tbad020_S2_tbad020

Fig_tbad020_S3_tbad020

Fig_tbad020_S4_tbad020

Fig_tbad020_S5_tbad020

Fig_tbad020_S6_tbad020

Fig_tbad020_S7_tbad020

Fig_tbad020_S8_tbad020

Fig_tbad020_S9_tbad020

## Data Availability

The datasets used and analysed during the current study will be available from the corresponding author on reasonable request.

## ANIMAL RESEARCH STATEMENT

All animal procedures were performed in compliance with Institutional Animal Care and Use Committee (IACUC) guidelines and approved by the Institutional Animal Care and Use Committee of Shanghai Model Organisms Center, China.
