# Supplementary figures and images for: IMM47, a humanized monoclonal antibody that targets CD24, exhibits exceptional anti-tumor efficacy by blocking the CD24/Siglec-10 interaction and can be used as monotherapy or in combination with anti-PD1 antibodies for cancer immunotherapy
Source: Antib Ther. 2023 Sep 9;6(4):240–52. doi: 10.1093/abt/tbad020 (PMC10576855; doi:10.1093/abt/tbad020)

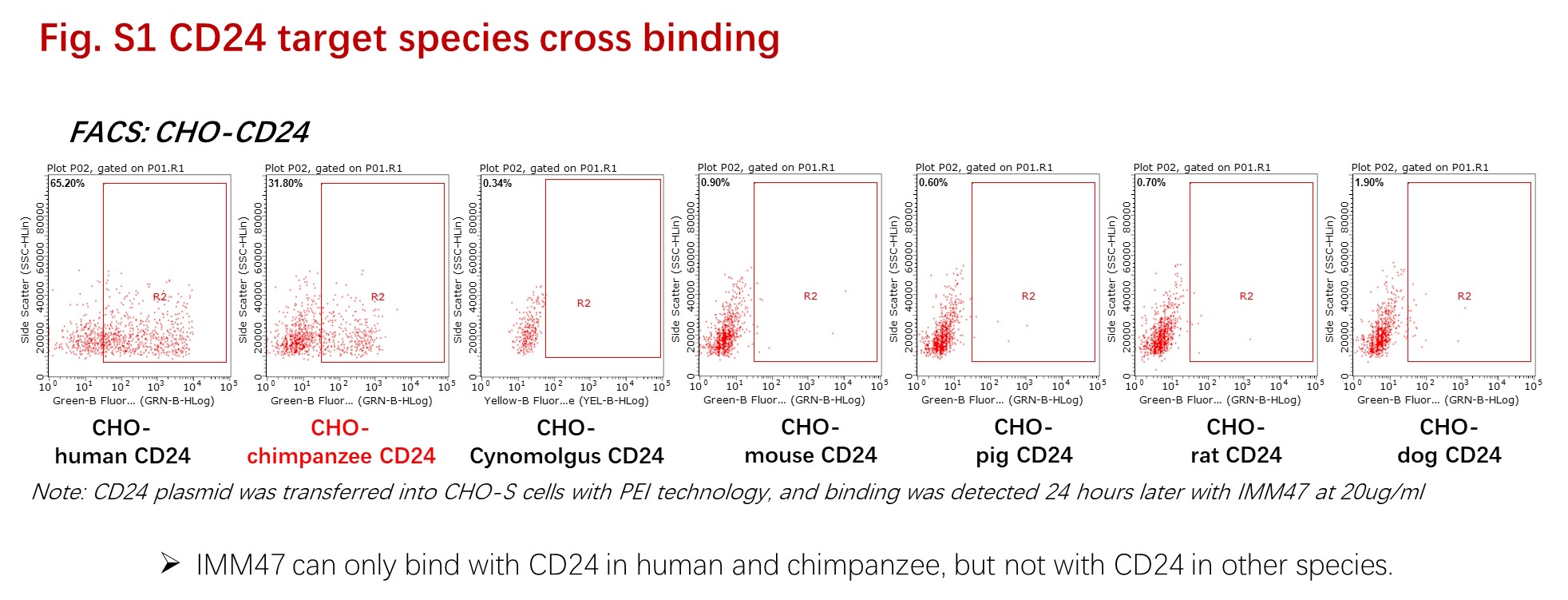

Supplement: Fig_tbad020_S1_tbad020 [file fig_tbad020_s1_tbad020.jpeg]

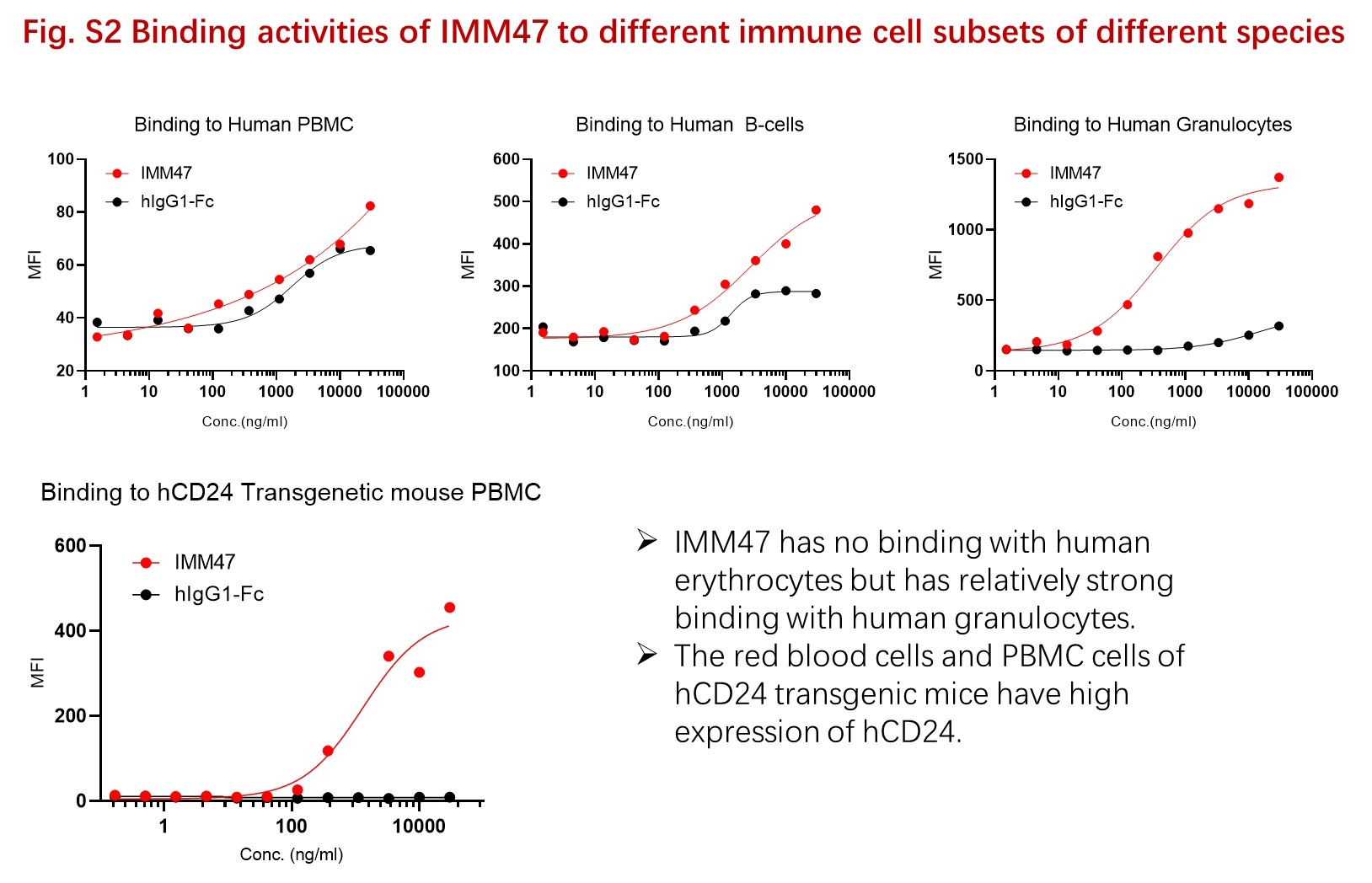

Supplement: Fig_tbad020_S2_tbad020 [file fig_tbad020_s2_tbad020.jpeg]

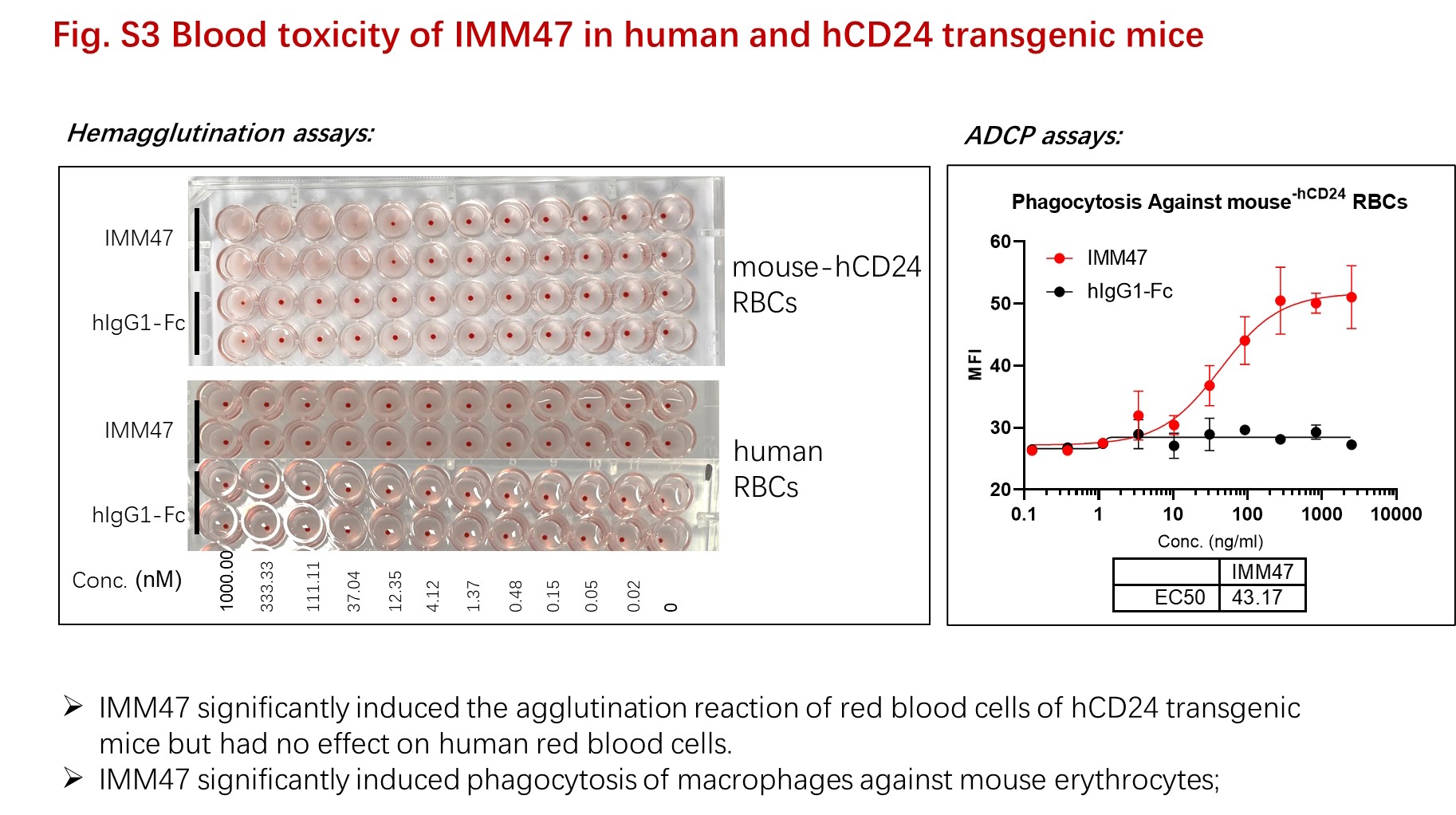

Supplement: Fig_tbad020_S3_tbad020 [file fig_tbad020_s3_tbad020.jpeg]

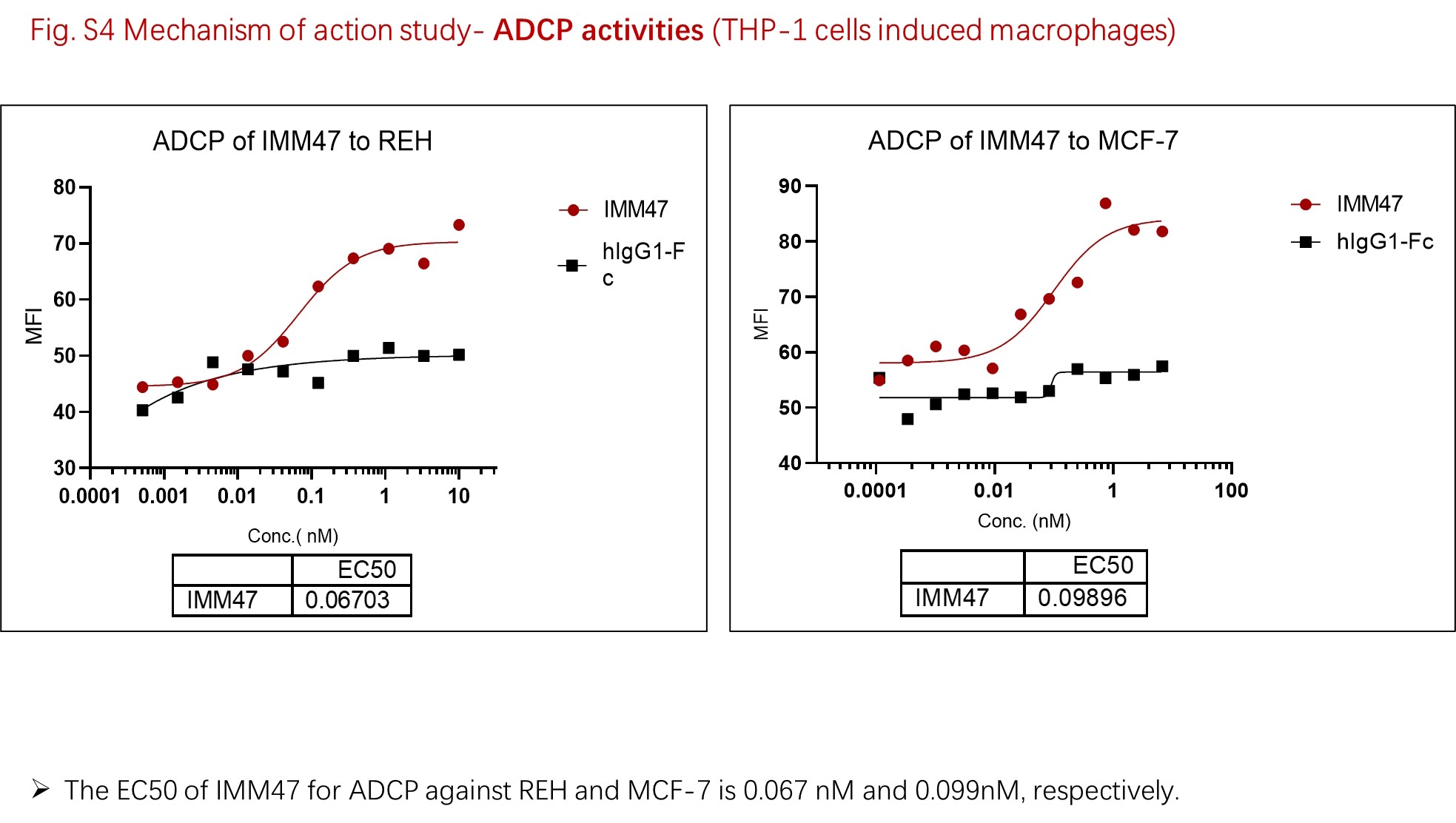

Supplement: Fig_tbad020_S4_tbad020 [file fig_tbad020_s4_tbad020.jpeg]

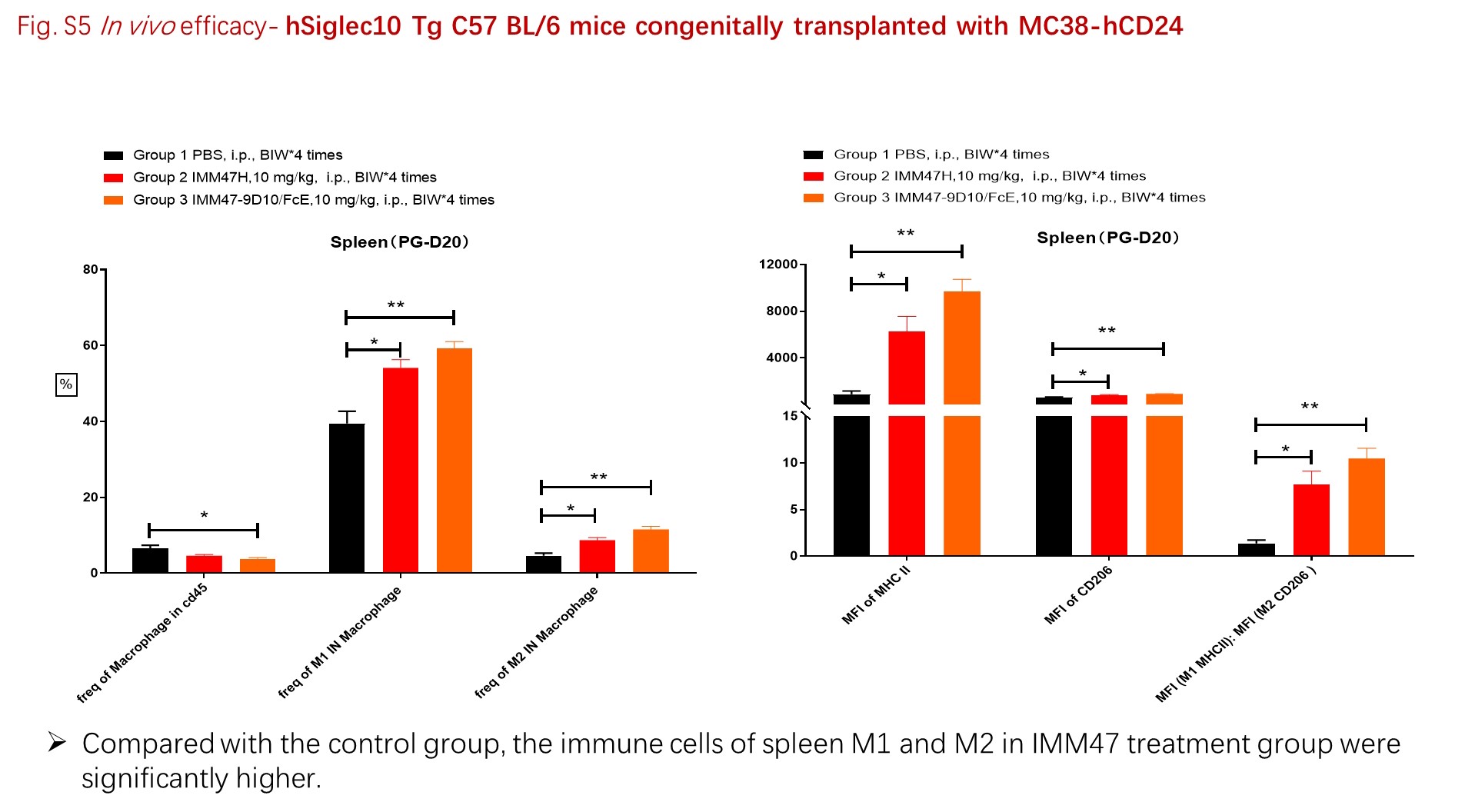

Supplement: Fig_tbad020_S5_tbad020 [file fig_tbad020_s5_tbad020.jpeg]

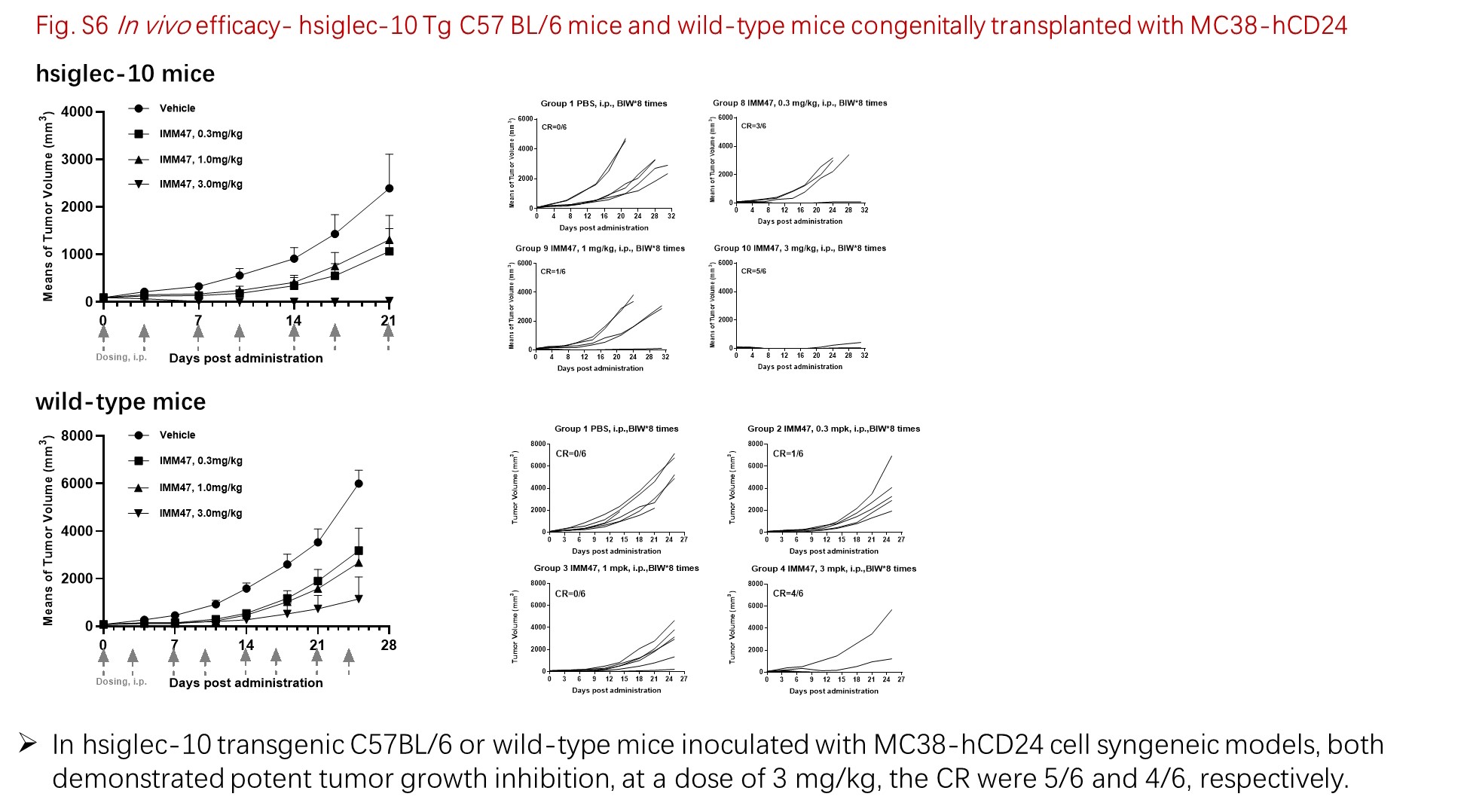

Supplement: Fig_tbad020_S6_tbad020 [file fig_tbad020_s6_tbad020.jpeg]

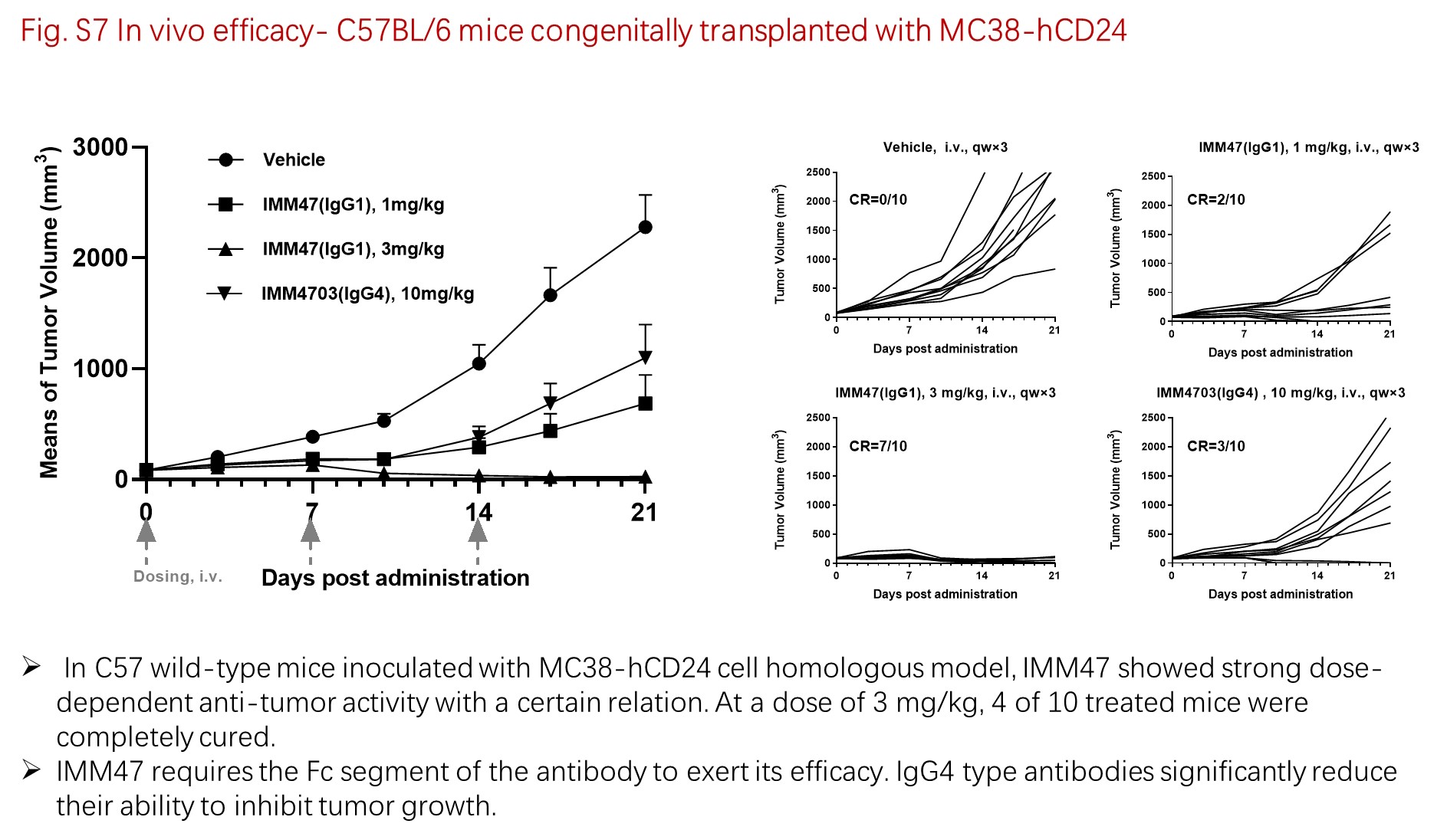

Supplement: Fig_tbad020_S7_tbad020 [file fig_tbad020_s7_tbad020.jpeg]

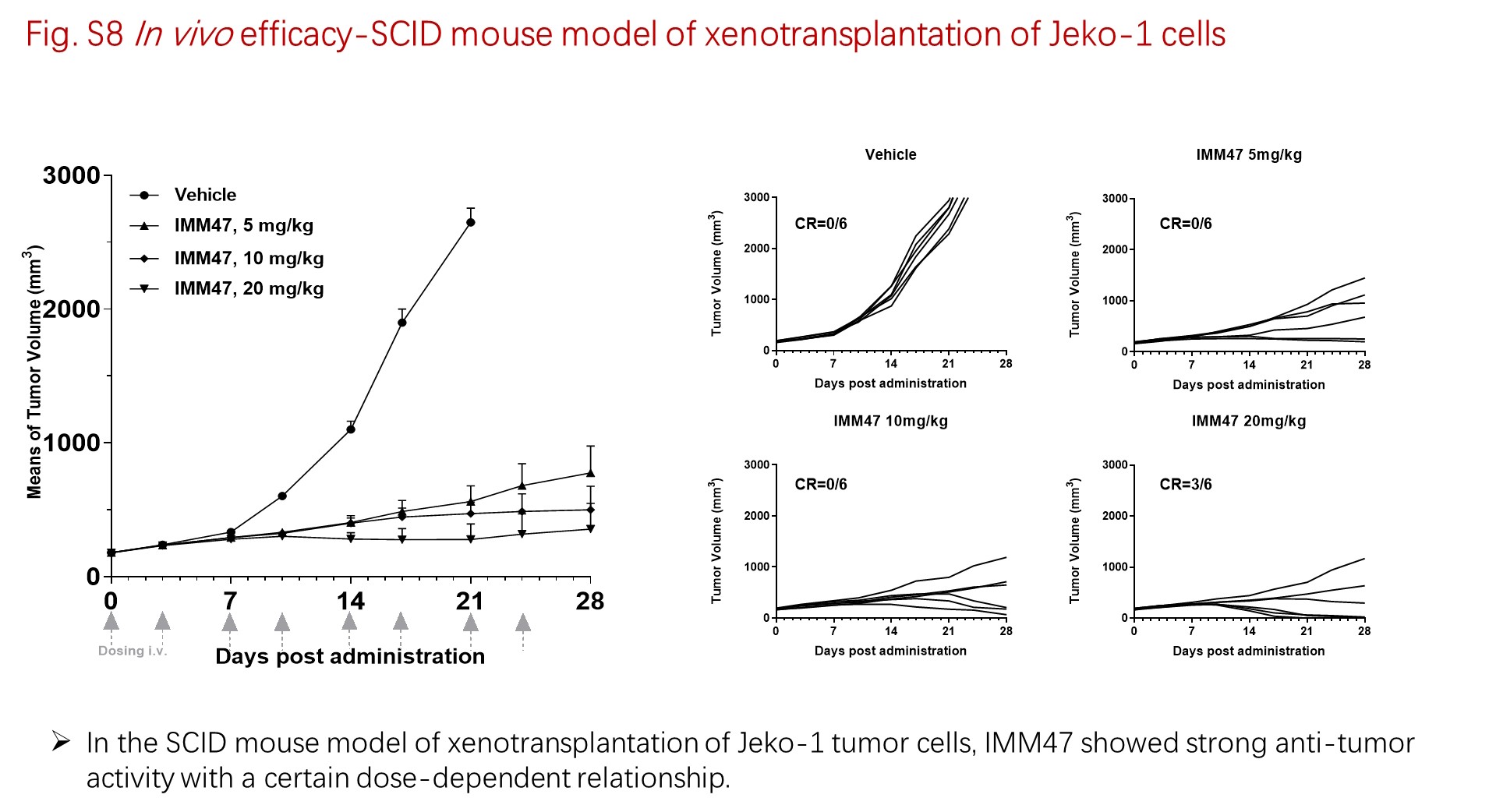

Supplement: Fig_tbad020_S8_tbad020 [file fig_tbad020_s8_tbad020.jpeg]

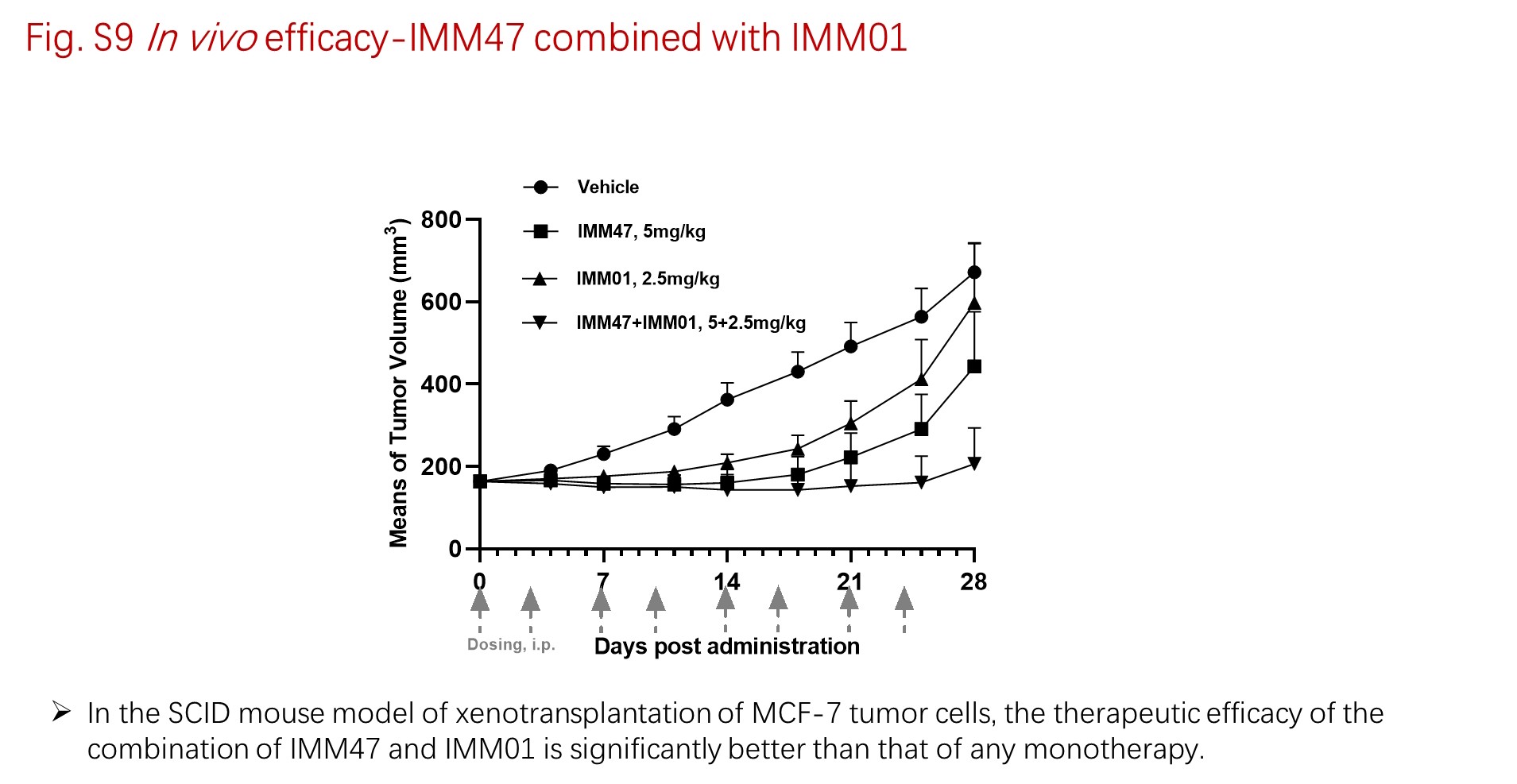

Supplement: Fig_tbad020_S9_tbad020 [file fig_tbad020_s9_tbad020.jpeg]
